# An Association Study of DNA Methylation and Gene Expression in Angelman Syndrome: A Bioinformatics Approach

**DOI:** 10.3390/ijms23169139

**Published:** 2022-08-15

**Authors:** Julia Panov, Hanoch Kaphzan

**Affiliations:** Laboratory for Neurobiology of Psychiatric Disorders, Sagol Department of Neurobiology, University of Haifa, Haifa 3498838, Israel

**Keywords:** Angelman syndrome, transcriptome, DNA methylation, RNA-seq, bisulfite-seq, multi-omics

## Abstract

Angelman syndrome (AS) is a neurodevelopmental disorder caused by the loss of function of the E3-ligase UBE3A. Despite multiple studies, AS pathophysiology is still obscure and has mostly been explored in rodent models of the disease. In recent years, a growing body of studies has utilized omics datasets in the attempt to focus research regarding the pathophysiology of AS. Here, for the first time, we utilized a multi-omics approach at the epigenomic level and the transcriptome level, for human-derived neurons. Using publicly available datasets for DNA methylation and gene expression, we found genome regions in proximity to gene promoters and intersecting with gene-body regions that were differentially methylated and differentially expressed in AS. We found that overall, the genome in AS postmortem brain tissue was hypo-methylated compared to healthy controls. We also found more upregulated genes than downregulated genes in AS. Many of these dysregulated genes in neurons obtained from AS patients are known to be critical for neuronal development and synaptic functioning. Taken together, our results suggest a list of dysregulated genes that may be involved in AS development and its pathological features. Moreover, these genes might also have a role in neurodevelopmental disorders similar to AS.

## 1. Introduction

Angelman syndrome (AS) is a human neuropsychiatric disorder associated with autism, intellectual disability, ataxia, sleep disturbances, lack of speech, and epilepsy [1,2]. In most cases, AS is caused by deletion of a small region of DNA on maternal chr15q11.2-q13 that includes the UBE3A gene [3,4,5,6]. Interestingly, the opposite molecular condition, Dup15q, where the UBE3A gene is duplicated on the maternal chr15q11.2-q13, is also characterized by autism, intellectual disability, hypotonia, language developmental delay and language deficits, and epilepsy [7,8]. In addition, the UBE3A gene has been shown to be associated with autism and schizophrenia [9,10].

The UBE3A gene encodes for the ubiquitin ligase E3A, also termed E6-associated protein (E6-AP). This protein is a HECT domain E3-ligase, a family of enzymes that covalently attaches ubiquitin chains to proteins, signaling them for degradation by 26S proteasome. In addition to the ligase activity of UBE3A, it has been shown that this protein has other functions that are not fully understood. There is evidence that UBE3A affects progesterone-receptor- and estrogen-receptor-dependent transcriptional activity [11,12,13,14]. Furthermore, it has been shown that loss of function of UBE3A leads to general genomic hypo-methylation [15,16]. However, our current understanding of molecular players and pathways affected by UBE3A expression fails to explain the full range of its interactions.

Multi-omics analysis is becoming increasingly popular in biomedical research. The unsupervised multi-layered molecular data allow researchers to infer significant molecular interactions and cascades associated with particular phenotypes with higher validity [17,18].

While multiple datasets have been derived from various models of AS, starting from fruit flies up to rodent models of mice and rats, only a few datasets have been obtained from neurons derived from human AS patients [19,20,21,22,23,24]. To date, no study has tried to associate different human-derived neuronal datasets using a multi-omics approach. In the current study, we transect publicly available human-derived neuronal transcriptome and DNA methylation datasets for multi-omics analyses and elucidation of the molecular mechanisms involved in AS.

## 2. Results

To elucidate the molecular mechanisms associated in AS with loss of function of the UBE3A protein, we analyzed the publicly available RNA sequencing (RNA-seq) and DNA methylation (bisulfite-seq) data of Angelman syndrome patients. The RNA-seq dataset was taken from the NCBI archive under the accession number PRJNA255989 [20]. The bisulfite-seq data were taken from the Gene Expression Omnibus dataset under accession number GSE8154157 [15]. The raw sequencing data were analyzed for enrichment of known molecular pathways differentially expressed and differentially methylated in AS patients compared to healthy controls.

**Figure 1:**
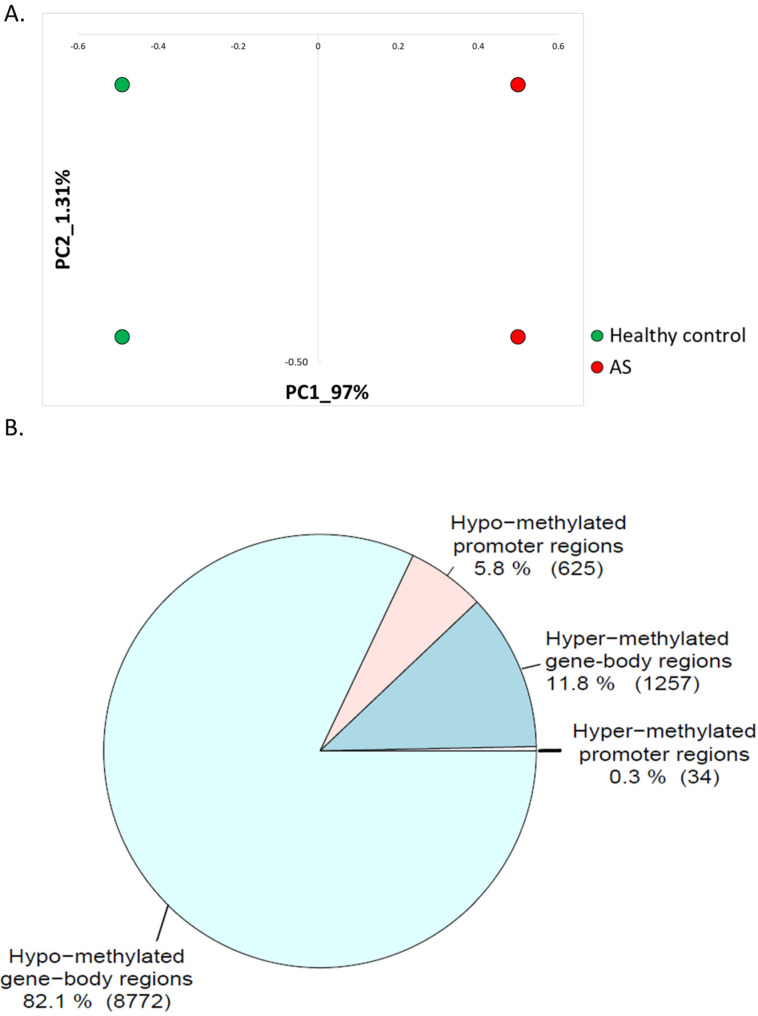
(**A**) Principal component analysis (PCA) of gene expression iPSC-derived neurons (PRJNA255989). (**B**) Pie chart showing the number of hyper-methylated regions near promoters (±1000 bp) (34 regions); hyper-methylated regions inside the gene bodies (1257 regions); hypo-methylated regions near promoters (±1000 bp) (625 regions); and hypo-methylated regions inside gene-bodies (8772 regions).

RNA sequencing raw reads were preprocessed to eliminate low-quality reads. The remaining reads were aligned to the assembled human genome (GRCh38.p12), and gene expression profiles were calculated. Principal component analysis (PCA) showed a clear separation between healthy controls and AS-derived iPSCs (Figure 1A). We found 209 upregulated genes in AS samples (Supplementary Materials, Table S1). Of these, 12 genes were highly expressed in AS samples and not expressed at all in control samples: COL22A1, EIF3CL, ERP27, EVA1A, FAM135B, GPR1, KIAA0040, KRT80, LYNX1, OLFML1, POTEI, and ZNF558. We also identified 31 downregulated genes in AS (Supplementary Materials, Table S2). Interestingly, 5 of these genes are associated with synaptic transmission: GABRA2, GAD1, SLC18A2, SST, and WNT7A.

The analysis of DNA methylation included per-position methylation calling based on Poisson distribution statistics (see Methods section). For a per-position contrast between scores in AS and healthy controls, the whole-genome profiles were segmented using the BinS algorithm [25,26], resulting in significantly differentiated hyper- and hypo- methylated regions in AS. The positions of the differentially methylated fragments of the genome were aligned with the positions of known promoter regulatory elements and with annotated genes.

We observed a high bias toward hypo-methylation of genome regions in AS. In addition, from a total of 10,688 differentially methylated regions aligned with promoter regions or gene bodies, 9397 (88%) were hypo-methylated in AS (Figure 1B).

We found 34 hyper-methylated regions of the genome that were located in proximity (±1000 bp) to gene promoter regions (Supplementary Materials, Table S3). Five of the hyper-methylated genes are known to regulate apoptosis: FANK1, NLRC4, ALDH1A2, LPAR1, and USP17L13. Two more genes (the KCNN2 gene that codes for the SK2 channel and LPAR1) are known to regulate neuronal spine development and are directly involved in learning, memory, and behavior regulation [27,28,29,30,31,32].

**Figure 2:**
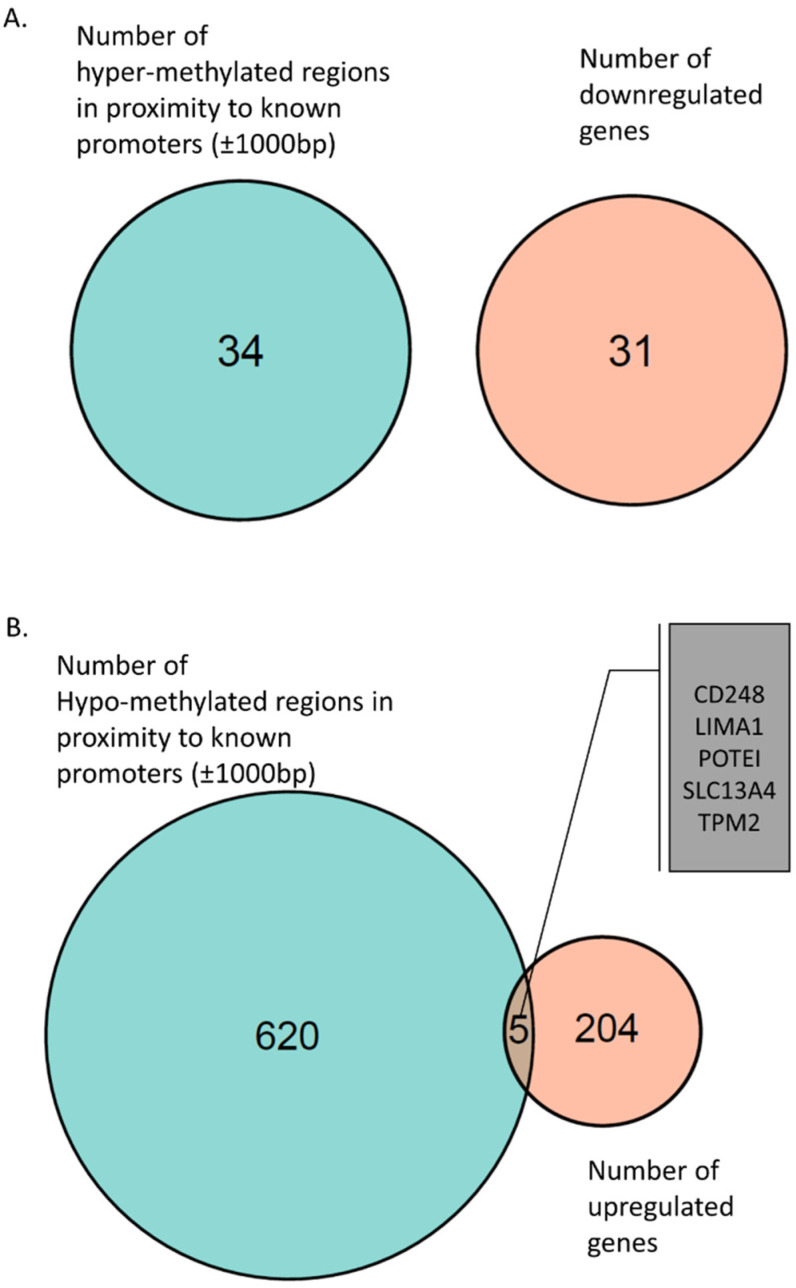
(**A**) Venn diagram of hyper-methylated regions near promoters (±1000 bp) and downregulated genes associated with these promoter regions. (**B**) Venn diagram of hypo-methylated regions near promoters (±1000 bp) and upregulated genes associated with these promoter regions. Five genes were found to be hypo-methylated and upregulated: CD248, LIMA1, POTEI, SLC13A4, and TPM2.

Hyper-methylation of promoter regions has been associated with downregulation of gene expression [33]. We investigated whether the genes associated with hyper-methylated promoters were differentially expressed in AS-patient-derived iPSCs. However, we did not find any downregulated genes associated with the hyper-methylated promoters (Figure 2A).

Next, we identified 625 hypo-methylated regions in proximity to known gene promoters (±1000 bp) (Supplementary Materials, Table S4). Hypo-methylated promoter regions are known to be “open” for transcription and thus are expected to be associated with upregulated expression of the genes [33]. We found five of these genes upregulated in iPSC-derived neurons from AS patients: POTEI, CD248, TPM2, LIMA1, and SLC13A4 (Figure 2B and Supplementary Materials, Table S5). Interestingly, the POTEI gene was found to have a hypo-methylated promoter region and was also expressed only in AS samples.

**Figure 3:**
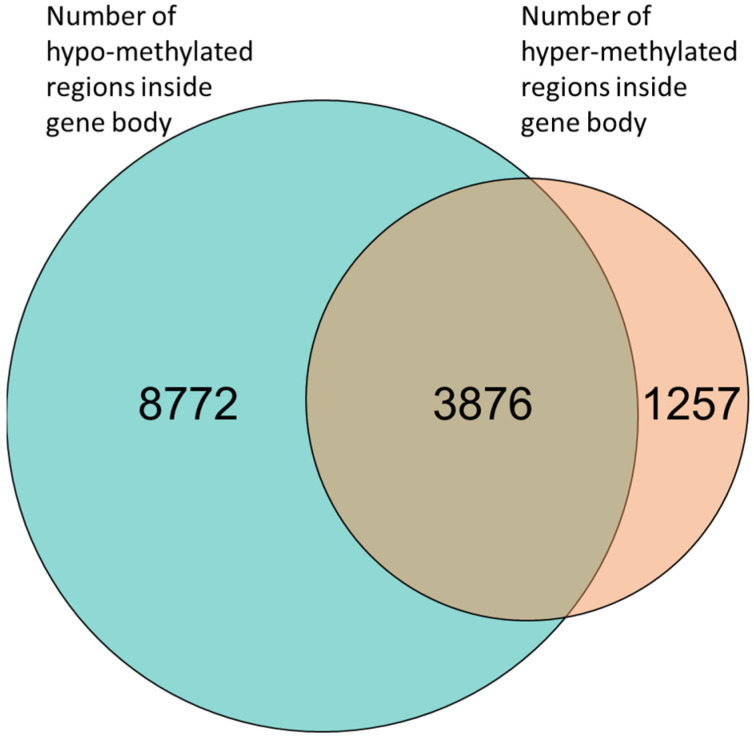
Venn diagram of gene-body methylation. Many genes (3876) had both hyper-methylated and hypo-methylated regions. However, 8772 genes had only hypo-methylation regions and 1257 genes had only hyper-methylated regions.

It has been shown that in addition to methylation of promoters, methylation of gene-body regions also has a regulatory effect on the expression of genes [34,35]. Nonetheless, it is still unclear whether methylation of the gene body represses or enhances expression [36,37,38]. Furthermore, we found that many of the genes contained both hyper- and hypo- methylated regions in their gene bodies (Figure 3). This confounding effect can be explained by the heterogeneity of the cells constituting the studied tissue. It is known that DNA methylation is cell-type specific, and thus different regulations can be present in different cells [39]. To avoid further perplexity, we investigated genes which had unique epigenetic signals in their gene body, i.e., either a hyper-methylated region or a hypo-methylated region.

**Figure 4:**
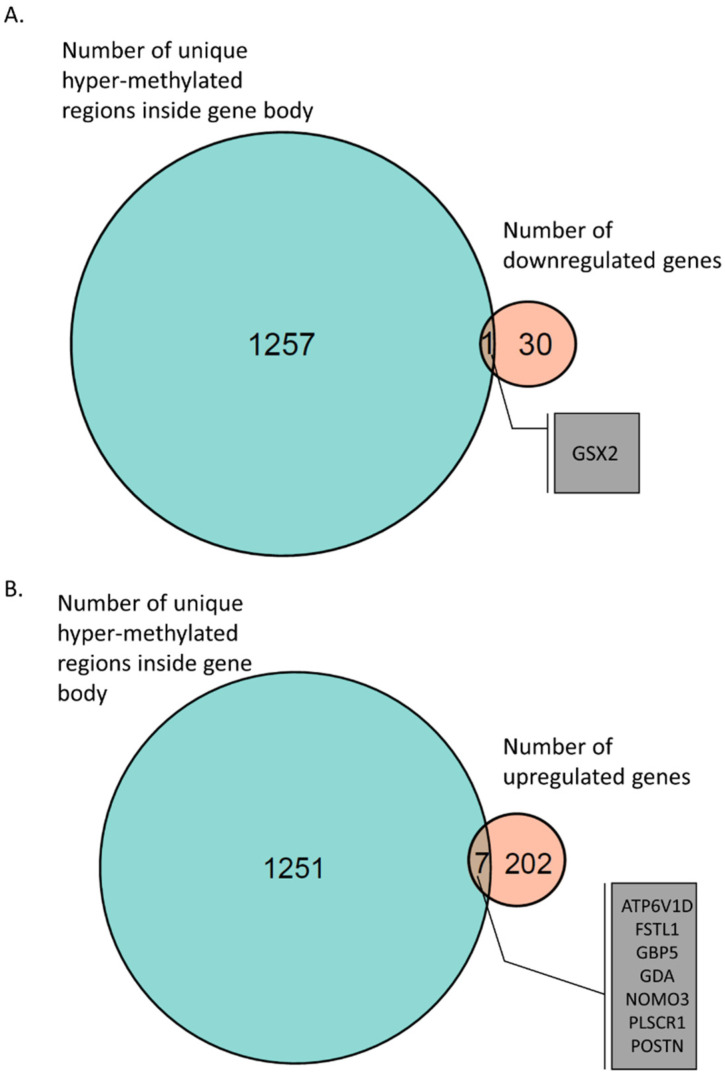
Venn diagram of gene-body hyper-methylation and gene expression. (**A**) GSX2 gene, which had a hyper-methylated region inside its gene body and was found to be downregulated in AS-derived iPSC neurons. (**B**) Seven genes were identified with hyper-methylated regions inside their gene bodies and were found to be upregulated in AS iPSC-derived neurons.

We found 1257 genes that had unique hyper-methylated regions in their gene body (Figure 3 and Supplementary Materials, Table S6). Further investigation of the expression of these genes showed that the GSX2 gene was downregulated in AS (Figure 4A). GSX2 is a transcription factor required for neuronal development [40,41,42,43]. In addition, we found 7 genes that were hyper-methylated in the gene-body regions, and their expression was upregulated in AS (Figure 4B and Supplementary Materials, Table S7).

We found 8772 genes that had a unique hypo-methylated region in their gene body (Figure 3 and Supplementary Materials, Table S8). This extraordinary high number of hypo-methylated genes (almost seven times as many genes had hypo-methylated regions than had hyper-methylated regions) suggests that overall, the genome in AS is hypo-methylated. This observation is in line with previous reports of overall DNA hypo-methylation in UBE3A knockdown cell lines [16]. Global hypo-methylation of DNA has been observed in many disorders and has been suggested to cause the genomic instability observed in many types of cancers [44,45] and in neuropsychiatric disorders [46,47].

**Figure 5:**
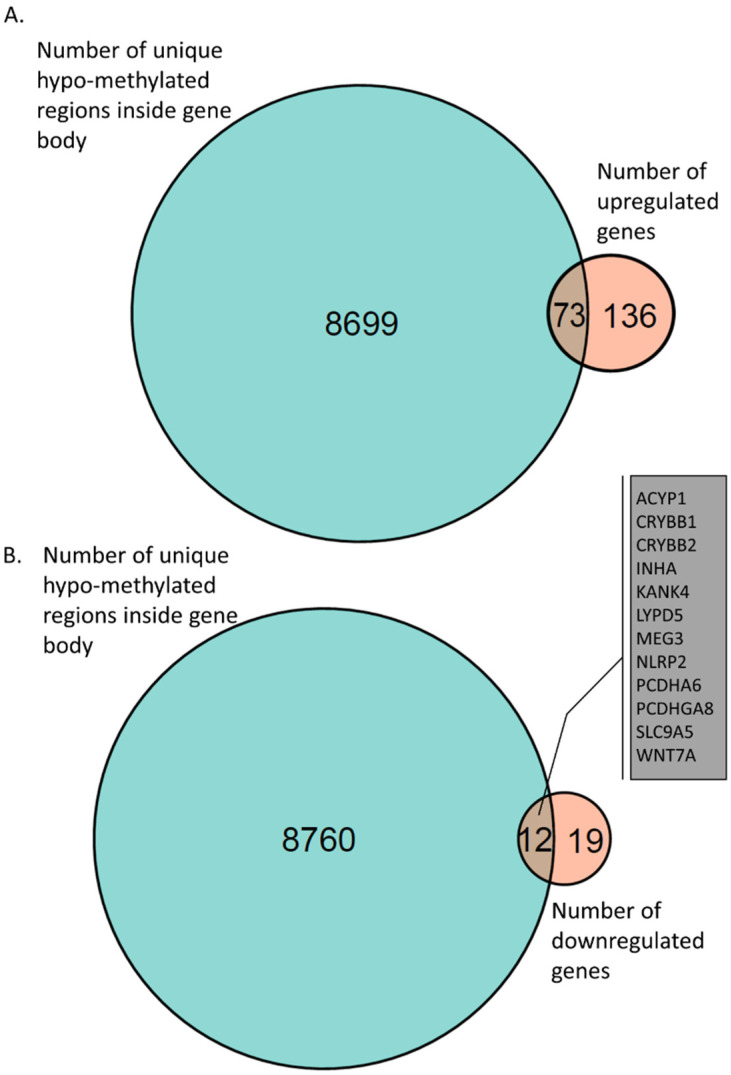
Venn diagram of gene-body hypo-methylation and gene expression. (**A**) A total of 73 genes were identified with a hypo-methylated region inside the gene body that were found in AS-derived iPSC neurons. (**B**) A total of 12 genes were identified with hypo-methylated regions inside their gene bodies that were found to be downregulated in AS-derived iPSC neurons.

We next investigated genes that were hypo-methylated in their gene-body regions and upregulated in iPSCs derived from AS patients. We found 73 such hypo-methylated and upregulated genes (Figure 5A and Supplementary Materials, Table S9). Of particular interest, were genes associated with synapse and neuronal activity, such as GPR176, RAB29, C1QL1, EXT1, GLRB, HRH1, HAPLN1, THBS2, and GPNMB. Furthermore, we identified 12 genes with hypo-methylated loci in their gene bodies that were downregulated in AS (Figure 5B and Supplementary Materials, Table S10).

**Figure 6:**
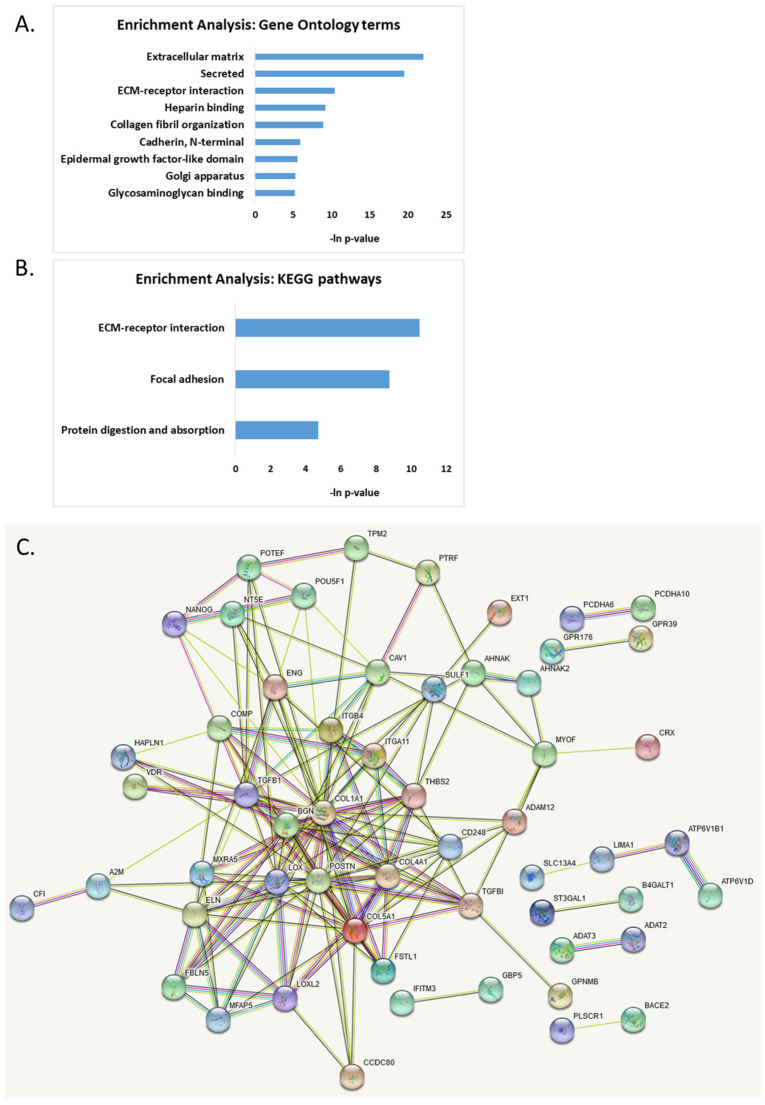
Functional enrichment analysis of genes found to be dysregulated both on DNA methylation level and on gene expression level. (**A**) Gene ontology enrichment analysis; (**B**) KEGG pathways enrichment analysis; (**C**) protein–protein interaction map generated with STRING tool.

Combining all aberrantly DNA-methylated and mRNA-dysregulated genes in AS, we found that they form several important functional clusters related to cell–cell adhesion (Figure 6A,B). These functional clusters included “extracellular matrix”, “ECM-receptor interaction pathway”, “secreted”, “heparin binding”, “collagen fibril organization”, “*N*-terminal cadherin”, and “glycosaminoglycan binding”. These pathways included 20 genes that were differentially methylated and expressed in AS samples compared to healthy controls (BGN, CCDC80, COL1A1, COL4A1, COL5A1, COMP, ENG, EXT1, FSTL1, GPNMB, ITGA11, ITGB4, LOXL2, PCDHA10, PCDHA6, PCDHGA8, PCDHGB6, POSTN, SULF1, and THBS2). We identified several transcription factors regulating the genes found as dysregulated in AS by utilizing the TRRUST database [48]: SP1, LMX1B, MYBL2, ZEB1, SNAI2, SF1 (Supplementary Materials, Table S11). Remarkably, the SP1 transcription factor, which is known to regulate 12 of the identified dysregulated genes (Supplementary Materials, Table S11), has been shown to be activated by UBE3A [12,49], and thus the downstream effect of dysregulation of SP1 may be observed in AS samples.

The protein–protein interaction analysis using STRING [50,51] revealed several hub proteins with more than 10 edges: COL1A1, POSTN, TGFB1, COL5A1, BGN, LOX, COL4A1, ELN, THBS2, CAV1, and TGFBI (Figure 6C).

## 3. Discussion

Multiple studies have used omics technologies in various biological models for Angelman syndrome [7,15,20,22,52,53,54,55,56,57], yielding multiple genes of interest. Herein, we attempt to associate two datasets obtained from neurons that were derived from human AS patients and healthy controls. The DNA methylation data were generated from postmortem brains of AS patients and healthy controls [15]. The mRNA sequencing data were generated from iPSC-derived neurons from AS patients and healthy controls [20]. We explored the interaction between the epigenetic level and the transcriptome level of cellular mechanisms, believing that the two datasets strengthen each other and that differentially expressed genes that are also found to have aberrant DNA methylation signals are strong candidates for the phenomenology of AS.

DNA methylation in the promoter region of genes is known to attenuate gene expression. However, the effect of DNA methylation inside the gene body on the expression is not clear. We identified hyper- and hypo-methylated regions by comparing methylation frequencies in two pairs of AS and control samples. If the methylation frequency was statistically higher in the AS samples than in the control samples, this region of the genome was called “hyper-methylated” in AS. When the methylation signal in control samples was higher than in AS, we called these regions “hypo-methylated” in AS. These found methylated regions were compared with genome annotation to identify the type of regulatory region, either a region close to the promoter (±1000 bp) or a differentially methylated region inside the gene body. Next, we identified differentially expressed genes in AS iPSCs-derived neurons. Finally, we could reveal which differentially expressed genes were also aberrantly methylated in AS. It is important to note that both DNA methylation and gene expression status show great variation among different cell types [58,59,60,61], and therefore, cell-type-specific or single-cell epigenetic analyses will also be important in the future.

Before implementing the crossing between the DNA methylation and mRNA expression we explored the gene expression data. Principal component analysis (PCA) showed that healthy control samples were clearly separated from AS samples (Figure 1A). We found 31 genes that were downregulated in AS and 209 (87%) genes that were upregulated in AS. Of most interest were genes with striking differences in their mRNA expression. We found 12 genes (COL22A1, EIF3CL, ERP27, EVA1A, FAM135B, GPR1, KIAA0040, KRT80, LYNX1, OLFML1, POTEI, and ZNF558) that were highly expressed in AS iPSCs and not expressed at all in control iPSC neurons (Supplementary Materials, Table S1). One of these genes was LYNX1, which is known to bind the extracellular face of the nicotinic receptors of the cholinergic system. LYNX1 is a critical modulator of memory, learning, and plasticity. It has been shown that the removal of LYNX1 in animal models leads to memory and plasticity enhancements [62]. Another gene that we found to be highly expressed in AS neurons and not expressed at all in control neurons, EVA1A1, is a key player in autophagy regulation [63]. EVA1A is essential for neuronal differentiation and neurogenesis [64,65]. Another upregulated gene was ERP27, which binds unfolded proteins. This could explain its role in AS, where the UBE3A gene is lacking. UBE3A, as an E3-ligase, is predicted to induce degradation of proteins by tagging them with ubiquitin chains. Some of the proteins that are marked for degradation are damaged, such as unfolded proteins. Hence, the lack of UBE3A in AS presumably causes a rise in the expression of unfolded proteins. Therefore, the upregulation of ERP27, which binds unfolded proteins, is reasonable. In addition, we found high expression of the GPR1 gene in AS and no detectable expression in control iPSCs. GPR1 shares sequence similarity (43%) with opioid neurotransmitters [66,67] and is known to regulate non-genomic estrogen effects [67].

Of the 31 downregulated genes, 4 were not expressed at all in AS iPSC samples (CRYBA4, FAM167B, GABRA2, and POU3F4) (Supplementary Materials, Table S2). Of these, the GABRA2 gene coding for the alpha-2 subunit of the GABAA receptor is of most interest. GABA is involved in balancing excitatory and inhibitory responses and is critical to proper brain functioning [68]. It has been shown that the expression of the GABRA2 gene is dysregulated in several neuropsychological conditions such as autism spectrum disorder [69], schizophrenia [70], depression [71], and impulsive behavior [72].

DNA methylation is a critical regulatory mechanism implicated in brain development, learning, memory, and disease in the human brain [73,74,75]. In contrast to gene expression, few if any DNA methylation differences among phenotypically normal human brain regions have been reported [76,77,78]. However, DNA methylation is known to be altered in patients with neuropsychiatric diseases, including schizophrenia [79], Alzheimer’s [80], and major depressive disorder [81].

Overall, we observed hypo-methylation of the AS genome (Figure 1B). We found that 9397 (88%) of the differentially methylated regions (both in proximity to promoters and inside gene bodies) were hypo-methylated in AS. Interestingly, overall hypo-methylation of the genome has been observed in many diseases, especially in different cancers [82,83].

Thirty-four (34) genes were found to be hyper-methylated in proximity (±1000 bp) to their promoter (Supplementary Materials, Table S3). Some of these hyper-methylated genes in AS are known to regulate apoptosis (FANK1, NLRC4, ALDH1A2, LPAR1, and USP17L13), which coincides with our previous finding that apoptosis is dysregulated in AS [53]. Two more genes (KCNN2 and LPAR1) regulate neuronal spine development, which is known to be dysregulated in AS. While the KCNN2 gene that codes for the SK2 channel is known to be dysregulated in AS [32,84,85], LPAR1 is known to be dysregulated in the AS-like Pitt–Hopkins syndrome [86]. Another important gene with a hyper-methylated promoter was PRKACG, which is known to repress pain sensation by regulating the transcription of the DREAM protein, coinciding with our previous observation that AS mice have disrupted pain perception [54]. In addition, the GIT2 gene promoter was found to be hyper-methylated in AS. GIT2 is known to be the main coordinator of aging processes, including obesity, which is one of the phenotypes of AS [87].

Hyper-methylation of the promoter region is known to repress gene expression; thus, we checked whether any of the 31 downregulated genes were among the genes with hyper-methylated promoters. We did not find any intersecting genes (Figure 2A).

We found 625 genes with a hypo-methylated region in proximity to their promoter (Supplementary Materials, Table S4). Hypo-methylated promoter regions are known to enhance gene expression; thus, we identified 5 genes that were upregulated in AS iPSCs (Figure 2B and Supplementary Materials, Table S5). One of these hypo-methylated and upregulated genes was the POTEI gene. The POTE (prostate-, ovary-, testis-, and placenta-expressed) family of genes has been shown to have low expression in normal somatic tissues but is highly expressed in various cancers [88,89]. Interestingly, the expression of the POTE family of genes has been associated with DNA hypo-methylation and activation of LINE1 elements. In fact, POTE genes themselves harbor LINE1 elements [90]. Activation of LINE1 in cancer leads to genomic instability and mutagenesis and has been shown to drive cancer progression. In addition, it has been suggested that activation of LINE1 elements is involved in autism and other neurodevelopmental disorders [91,92]. Another gene amongst those that were hypo-methylated near their promoter region and highly expressed in AS was the SLC13A4 gene. SLC13A4 encodes for a sulfate transporter. Dysregulated SLC13A4 expression has been observed in several neurological and neurodevelopmental disorders and during seizures [93,94], which display shared phenotypes with AS patients.

In addition to methylation of promoter regions, it has been shown that methylation of the gene body has a regulatory effect on the expression of genes [35,45]. Nonetheless, this effect on the expression of genes is still not well understood; it is not clear whether gene-body methylation blocks or enhances gene transcription [36,37,38]. Hence, we divided our analyses of gene-body methylation into four associations with gene expression data. First, we considered genes hyper-methylated in their gene body and downregulated in AS samples. In the next association, we crossed the genes with hyper-methylated gene bodies with upregulated genes. Next, the same two types of associations were performed for hypo-methylated genes, i.e., genes that were hypo-methylated in their gene body and upregulated and hypo-methylated gene-body genes that were downregulated in AS samples.

Inside gene bodies, we found 5133 regions that were hyper-methylated in AS and 12,648 hypo-methylated regions. However, 3876 genes had both hyper- and hypo-methylated regions in AS (Figure 3). Thus, we further investigated only genes that had unique methylation status in AS, i.e., either hyper-methylated (1257, Supplementary Materials, Table S6) or hypo-methylated (8772, Supplementary Materials, Table S8).

The only gene with hyper-methylation of the gene-body region in AS and downregulated mRNA expression was the GSX2 gene (Figure 4A). GSX2 is a transcription factor required for neuronal development [40,41,42,43], and thus its hyper-methylation and downregulation is of high importance. In addition, we found seven hyper-methylated and overexpressed genes (Supplementary Materials, Table S7 and Figure 4B). One of these hyper-methylated and overexpressed genes was GDA, which codes for the protein Cypin. Cypin is important for neuronal development and is a regulator of PSD-95 postsynaptic signaling. It has been shown that overexpression of Cypin in cultured hippocampal neurons disrupts the synaptic clustering of PSD-95 and SAP-102 synaptic proteins.

We identified 8772 genes that had only a hypo-methylated signal in AS inside their gene bodies (Supplementary Materials, Table S8). Crossing these genes with upregulated genes in AS, we found 73 genes (Supplementary Materials, Table S9 and Figure 5A). Of special interest were genes associated with synapse and neuronal activity, such as GPR176, RAB29, C1QL1, EXT1, GLRB, HRH1, HAPLN1, THBS2, and GPNMB. Previously, it has been noted that synaptic functioning is dysregulated in AS [95,96,97,98,99]. Another interesting cluster of genes were the 17 genes known to be involved in cell adhesion: ADAM12, AJAP1, B4GALT1, COMP, COL1A1, COL5A1, ENG, GPNMB, HAPLN1, ITGA11, ITGB4, IL32, LOXL2, PCDHA10, PCDHGB6, THBS2, and TGFBI. Crossing the hypo-methylated regions with the downregulated genes we found 12 genes (Supplementary Materials, Table S10 and Figure 5B). The most significantly downregulated gene was MEG3, which is a long non-coding RNA previously implicated in Alzheimer’s pathology and in several cancers. MEG3 has been shown to be a powerful cell growth suppressor regulating the PI3K/Akt signaling pathway [100,101]. In addition, we found hypo-methylation and downregulation of two crystalline genes, CRYBB1 and CRYBB2, which were previously found to be associated with schizophrenia and autism-like behavior [102,103].

Finally, combining all of the above genes dysregulated on both methylation and expression levels in AS, we found several functional clusters dysregulated in AS (Figure 6A,B). Extracellular matrix (ECM) receptor interaction was one of the most significantly dysregulated pathways in AS. ECM receptors and their ligands play key roles in neuronal differentiation, communication, and synapse connection. They regulate synaptic activity and neuronal structure and function, and thereby affect animal behavior [104,105]. Recently, dysregulation of cell adhesion molecules in UBE3A-silenced cells revealed that these cells have impaired morphological development and pathway activation, leading to a delayed adhesion and defective contact guidance in response to stimuli [104].

Fascinatingly, the transcription factors that control the expression of the dysregulated genes in AS on both the DNA methylation level and on the mRNA expression level (Supplementary Materials, Table S11) included SP1 transcription factor. SP1 has been shown to be activated by UBE3A [12,49], and thus the downstream effect of dysregulation of SP1 may be observed in AS samples.

We also found that two of the genes found to be differentially expressed and differentially methylated, MEG3 and NLRP2, are maternally imprinted genes.

The protein–protein interaction analysis revealed 11 hub proteins with more than 10 edges: COL1A1, POSTN, TGFB1, COL5A1, BGN, LOX, COL4A1, ELN, THBS2, CAV1, and TGFBI (Figure 6C).

It is important to note that the results presented in our study should be considered with caution due to the small sample size both in the transcriptome dataset and the DNA methylation dataset. In effect, our study emphasizes the need for producing additional datasets to elucidate the molecular effect of UBE3A deletion in different cells and tissues.

To conclude, in this study, we observed that while the amount of correspondence between the epigenomic level of DNA methylation and the level of mRNA expression is not high, still there is some congruity between these two levels, which yields a list of genes that are significant for brain development. This list can serve future studies as a basis for further exploration of target genes that are involved in the pathophysiology of Angelman syndrome and other similar neurodevelopmental disorders.

## 4. Materials and Methods

### 4.1. RNA Sequencing Analysis

Raw RNA sequencing data were downloaded from the NCBI database (SRP04474952) [20]. For our analysis we used four samples from this project: two biological replicates of AS patient (del 1-0) iPSC-derived neurons (SRR1523347, SRR1523349) and two healthy control samples from Nml (1-0) iPSC-derived neurons (SRR1523352, SRR1523353). The AS patient iPSC-derived neurons were originally reported by Chamberlain et al. [105] and were generated from fibroblasts of patient with 15q11-q13 deletion. Raw sequencing reads were cleaned from adapter sequences using the Trimmomatic algorithm [106] and aligned to the human reference genome (GRCh38.p12) using TopHat2 [107]. Differentially expressed genes were identified with EdgeR [108]. We considered genes to be differentially expressed and upregulated in AS if they had a *p*-value < 0.005 and a fold change > 1.5. We considered genes to be differentially expressed and downregulated in AS if they had a *p*-value < 0.005 and a fold change < 0.6.

### 4.2. DNA Methylation Analysis

Whole-genome DNA methylation bisulfite sequencing data were downloaded from the NCBI database (GSE8154157) [15]. The dataset used for the analysis includes four samples: two AS patient samples, male and female (GSM2156992 and GSM2156993), and two healthy control samples, male and female (GSM2156974 and GSM2156973) of a relatively similar age (AS male age 23, AS female age 43, healthy control female age 42, and healthy control male age 42). All AS patients had maternal deletion of 15q11.2–q13.3 [15]. Raw reads were aligned to the human reference genome (GRCh38.p12) using the segemehl alignment algorithm with the [−F, −bisulfite 1] option [109]. Per-nucleotide methylation levels and differentially methylated regions were determined similarly to the procedure for identifying mutational frequencies in Li et al. [110].

Briefly, the frequency of each of the methylated cytosines across the genome in each sample was determined by the number of reads with C > T variant alignment on the positive strand and A > G variant alignment on the negative strand divided by the overall coverage of the genome at the given position. The accuracy of estimation of variant frequency can be influenced by the coverage of this position. Thus, to estimate the significance of the frequency of each variant we calculated the 95% confidence interval for the frequency of every variant at the given position from Wilks’ theorem for binomial distributions. Each frequency, with its confidence interval margins, was transformed to values of the quasi-normally distributed variable using the Fisher transformation:

$$v=ln\frac{\left(1+f\right)}{\left(1-f\right)}$$

The standard deviation (SD) of the variant frequency was evaluated as 1/6 of the confidence interval length after transformation of its margins.

After calculating the frequencies and the 95% confidence intervals of the methylated variant in each sample, we calculated the significance of the slope (*b*1 coefficient) of the linear regression between two pairs of frequencies in each methylated position (control male sample against AS male sample and control female sample against AS female sample):

Y=X×b+ε

where *b* = (*b*0, *b*1) is a vector column of parameters.

Vector *Y* consists of sub-columns (*Y*1, *Y*2) of the Gaussianized frequency observations in two samples (control and AS). Matrix *X* consists of two columns: a column of intercepts and a column consisting of −1 and +1 sub-columns matching the *Y*1 and *Y*2 sub-columns. The least squares estimation of vector *b* of parameters *b*0 and *b*1 is:

$$\hat{b}={\left(X^{T}X\right)}^{-1}X^{T}Y$$

This linear estimation gives a standard deviation for both parameter estimations, particularly SD(*b*1) for the *b*1 estimation that is a function of the SDs of *Y*(*i*) in the two groups, i.e., *b*1 is a linear combination of *Y*1(*i*) and *Y*2(*j*) stochastic variables. Therefore, assuming the independence of all *Y*(*i*) variables, SD(*b*1) is calculated via SD(*Y*(*i*)).

Based on SD (*b*1), the significance of *b*1 is calculated as the z-score of the fitness parameter *b*1:

*Y* = *b*0 + *b*1 × Group Difference

where *Y* (normalized frequency of methylation) is observed in two groups of samples with standard deviations SD1 and SD2 for *Y*1 and *Y*2 observations. The maximum likelihood (least squares) estimation of *b*1 gives the “slope” of the methylation—hyper-methylation (positive slope between control and AS groups) or hypo-methylation (negative slope between control and AS groups). The *b*1 estimation is also a normally distributed stochastic variable with its own SD that defines the significance (z-score) of the methylation *b*1.

Using the BinS algorithm [26] for genome segmentation, we found regions of the genome enriched by differential methylation signals in two pairs of samples (control male versus AS male and control female versus AS female). These segments were aligned to the known regulatory regions of the genome (promoter regions ± 1000 bp and gene bodies). If some regulatory region was found to be hypo- or hyper-methylated in both male and female pairs of samples, it was considered to be differentially methylated.

### 4.3. Enrichment Analysis

Gene enrichment analysis was performed using the DAVID [111,112,113] and Enrichr [114,115] web servers. The protein–protein interaction analysis was performed using STRING [50].

## Data Availability

The RNA-seq dataset was taken from the NCBI archive under the accession number PRJNA255989. The bisulfite-seq data were taken from the Gene Expression Omnibus dataset under accession number GSE8154157.

## Supplementary Materials

The following are available online at https://www.mdpi.com/article/10.3390/ijms23169139/s1.

## Acronyms and Abbreviations

AS	Angelman syndrome
PCA	principal component analysis
ALDH1A2	aldehyde dehydrogenase 1 family member A2
BGN	biglycan
C1QL1	complement C1q like 1
CAV1	caveolin 1
CCDC80	coiled-coil domain containing 80
CD248	CD248 molecule
COL1A1	collagen type I alpha 1 chain
COL22A1	collagen type XXII alpha 1 chain
COL4A1	collagen type IV alpha 1 chain
COL5A1	collagen type V alpha 1 chain
COMP	cartilage oligomeric matrix protein
EIF3CL	eukaryotic translation initiation factor 3 subunit C like
ELN	elastin
ENG	endoglin
ERP27	endoplasmic reticulum protein 27
EVA1A	eva-1 homolog A, regulator of programmed cell death
EXT1	exostosin glycosyltransferase 1
FAM135B	family with sequence similarity 135 member B
FANK1	fibronectin type III and ankyrin repeat domains 1
FSTL1	follistatin like 1
GABRA2	gamma-aminobutyric acid type A receptor subunit alpha2
GAD1	glutamate decarboxylase 1
GLRB	glycine receptor beta
GPNMB	glycoprotein nmb
GPNMP	glycoprotein nonmetastatic melanoma protein B
GPR1	chemerin chemokine-like receptor 2
GPR176	G protein-coupled receptor 176
GSX2	GS homeobox 2
HAPLN1	hyaluronan and proteoglycan link protein 1
HRH1	histamine receptor H1
ITGA11	integrin subunit alpha 11
ITGB4	integrin subunit beta 4
KCNN2	potassium calcium-activated channel subfamily N member 2
KRT80	keratin 80
LIMA1	LIM domain and actin binding 1
LMX1B	LIM homeobox transcription factor 1 beta
LOX	lysyl oxidase
LOXL2	lysyl oxidase like 2
LPAR1	lysophosphatidic acid receptor 1
LYNX1	Ly6/neurotoxin 1
MYBL2	MYB proto-oncogene like 2
NLRC4	NLR family CARD domain containing 4
OLFML1	olfactomedin like 1
PCDHA10	protocadherin alpha 10
PCDHA6	protocadherin alpha 6
PCDHGA8	protocadherin gamma subfamily A, 8
PCDHGB6	protocadherin gamma subfamily B, 6
POSTN	periostin
POTEI	POTE ankyrin domain family member I
RAB29	RAB29, member RAS oncogene family
SF1	splicing factor 1
SLC13A4	solute carrier family 13 member 4
SLC18A2	solute carrier family 18 member A2
SNAI2	snail family transcriptional repressor 2
SP1	Sp1 transcription factor
SST	somatostatin
SULF1	sulfatase 1
TGFB1	transforming growth factor beta 1
TGFBI	transforming growth factor beta induced
THBS2	thrombospondin 2
TPM2	tropomyosin 2
UBE3A	ubiquitin protein ligase E3A
USP17L13	ubiquitin specific peptidase 17 like family member 13
WNT7A	Wnt family member 7A
ZEB1	zinc finger E-box binding homeobox 1
ZNF558	zinc finger protein 558
